# Incidence and determinants of severe maternal morbidity: a transversal study in a referral hospital in Teresina, Piaui, Brazil

**DOI:** 10.1186/s12884-015-0648-3

**Published:** 2015-09-07

**Authors:** Alberto Pereira Madeiro, Andréa Cronemberger Rufino, Érica Zânia Gonçalves Lacerda, Laís Gonçalves Brasil

**Affiliations:** Research Center and Extension Center in Women’s Health, Piaui State University, R. Olavo Bilac, 2335 – Centro/Sul, Teresina, Piauí CEP 64001-120 Brazil; Av. Coronel Costa Araújo, 3033, Teresina, Piauí 64049-460 Brazil

## Abstract

**Background:**

Maternal near miss (MNM) investigation is a useful tool for monitoring standards for obstetric care. This study evaluated the prevalence and the determinants of severe maternal morbidity (SMM) and MNM in a tertiary referral hospital in Teresina, Piauí, Brazil.

**Methods:**

A transversal and prospective study was conducted between September 2012 and February 2013. The cases were included according to criteria established by the WHO. Odds ratio, their respective confidence intervals, and multivariate analyses were examined.

**Results:**

Five thousand eight hundred forty one live births, 343 women with SMM, 56 cases of MNM, and 10 maternal deaths were investigated. The rate for severe maternal outcomes was 11.2 cases per 1000 live births, the rate of MNM was 9.6 cases/1000 live births, and the rate for mortality was 171.2 cases/100,000 live births. Management criteria were most frequently observed among MNM/death cases. Hypertensive diseases (86.1 %) and hemorrhagic complications (10.0 %) were the main determinants of MNM, but infectious abortion was the most common isolated cause of maternal death. There was a correlation between MNM/death and hospitalized more than 5 days (*p* = 0.023) and between termination of pregnancy by cesarean (*p* = 0.002) and APGAR < 7 in the 1^st^ minute (*p* = 0.015).

**Conclusions:**

SMM and MNM were quite prevalent in the population studied. Women whose condition progressed to MNM/death had a higher association with terminating pregnancy by cesarean, longer hospitalization times, and worse perinatal results. The results from the study can be useful to improve the quality of obstetric care and consequently diminish maternal mortality in the region.

## Background

Despite being more common in low-income countries, maternal deaths are still a relevant public health problem among middle-income countries. There is a great disparity in the ratio of maternal mortality among countries, varying from less than 10 deaths per 100,000 live births in developed regions to 1200/100,000 live births in low-income regions [1]. In Brazil the ratio of maternal mortality was 64.8/100,000 live births in 2011 [2], with worse indicators in the North (77.8/100,000 live births) and Northeast (80.8/100,000 live births) regions. The states of Maranhão (114.0/100,000 live births) and Piauí (101.8/100,000 live births) had highest ratios in the country [3]. Hypertensive diagnoses, hemorrhagic disorders, complications during delivery, and abortion are among the principal causes of death among Brazilian pregnant women [4].

The study of maternal mortality is a challenge for researchers and governmental agencies mainly due to a lack of reliable data. Problems such as incomplete coverage of the information system and incomplete death certificates are common in Brazil [3]. Despite to provide useful information as a measure of obstetrical care, the small number of deaths in each institution limits the use of maternal mortality indicators for surveillance of maternal health in pregnancy [5, 6]. The search for a new indicator that could contribute to monitoring and evaluating maternal health brought about the development of the concept of severe maternal morbidity or near miss [7].

The term near miss was used for the first time in 1991 to refer to women who almost died from complications during the pregnancy-childbirth cycle, but who survived by chance or due to heath care they received [8]. Severe maternal morbidity represents the occurrence of a complication that could progress to maternal death, located in a spectrum between healthy pregnancy and maternal death [9]. The terms “near miss” and “severe maternal morbidity” are used interchangeably, but severe maternal morbidity reflects a less serious condition and represent a situation that precedes near miss cases in severity [10]. In the majority of cases, near miss has the same determinants as cases that end in death. Due to its greater frequency, the investigation of near miss cases allows for better and quicker understanding of the sequence of errors that lead to maternal death [11].

In the last few decades, the concept and the validity of maternal near miss have been gradually established, but controversies still exist about the criteria used to define the cases. In 2009 the World Health Organization (WHO) suggested that the investigation of the cases of near miss be part of reference monitoring used in reproductive health. Also, a research group standardized diagnostic criteria for this situation. According to these criteria, the emphasis should be focused on organic failure or dysfunction, which is identified using three categories: clinical, laboratory, and management [12, 13].

The prevalence of maternal near miss is variable around the world. Data from a systematic review of 46 countries demonstrate that the prevalence varies from 0.04 to 14.98 %, with higher rates in lower income regions in Africa and Asia [14]. A study in Holland in 2010, which evaluated 358,874 births, observed 2.4 cases of maternal near miss per 1000 live births [15]. In Brazil a multicentric study of 27 maternity reference centers, which utilized WHO criteria to identify near miss cases, registered 3.5 cases per 1000 live births [16]. Older pregnant women [17] and those with hypertensive syndromes [18] were more associated with maternal near misses.

Estimations of maternal near misses are complicated in a large country like Brazil. It is possible that the incidence and the determinants are not homogeneous among the regions that have diverse socioeconomic contexts, especially among the poorer regions. The metropolitan area of Teresina, in Piauí, Brazil, has one of the lowest indices of human development among major Brazilian cities and presents elevated rates of maternal mortality. Using WHO criteria, the present study investigated the incidence and the determinants of severe maternal morbidity and maternal near miss in a public tertiary referral maternity hospital in Teresina, where data on the topic are inexistent.

## Methods

### Design and location of the study

A transversal study was conducted from September 1, 2012 to February 28, 2013 to identify cases of severe maternal morbidity and maternal near miss at the Dona Evangelina Rosa Maternity in Teresina, in the Northeast of Brazil. This is the only tertiary obstetric hospital in the state of Piauí and surrounding area, providing care for the middle and low income population that depends on the public health care system. It is responsible for around 1200 monthly admissions, 1000 of which are for deliveries.

### Case selection

Four trained researchers identified cases on a daily basis by actively searching through medical files. All patients who presented complications during pregnancy and delivery, up to 42 days postpartum, according to the definitions provided by the WHO Research Group on Maternal Morbidity and Mortality were included in the study [12]. The cases of severe maternal morbidity included life-threatening conditions, such as serious postpartum hemorrhage, severe pre-eclampsia, eclampsia, and uterine rupture. Among the cases of severe maternal morbidity, those that included at least one of the clinical, laboratory, or management criteria were characterized as near miss [12]. Table 1 presents the inclusion criteria. Two control cases were selected to one severe maternal morbidity/ near miss cases, using simple random. All patients admitted on the same day of the cases and who did not fulfil the criteria for severe maternal morbidity/near miss cases were eligible to be controls. Patients were excluded from the consideration of criteria for near miss or severe morbidity in the case of maternal death. Sociodemographic information and information related to the pregnancy, in addition to perinatal outcomes, were collected by direct case record review using a form, which had been developed and pre-tested for this study, containing 36 codified questions.

**Table 1:** Criteria to identify potentially life-threatening conditions and maternal near-miss^a^

Severe maternal complications
1. Severe postpartum hemorrhage
2. Severe pre-eclampsia
3. Eclampsia
4. Sepsis or severe systemic infection
5. Ruptured uterus
6. Severe complications of abortion
Critical interventions or intensive care unit use
1. Admission to intensive care unit
2. Interventional radiology
3. Laparotomy (includes hysterectomy, excludes caesarean section)
4. Use of blood products
Near-miss criteria
A. Clinical organ dysfunction
1. Acute cyanosis
2. Gasping
3. Respiratory rate higher than 40 or lower than 6 bpm
4. Shock
5. Cardiac arrest
6. Oliguria non-responsive to fluids or diuretics
7. Any loss of consciousness lasting for more than 12 h
8. Stroke
9. Uncontrollable fit or status epilepticus
10. Global paralysis
11. Jaundice in the presence of pre-eclampsia
B. Laboratory markers
12. O_2_ saturation less than 90 % for more than 60 min
13. PaO_2_/FiO_2_ less than 200 mmHg
14. Creatinine more than 3.5 mg/dL
15. Bilirubin more than 6.0 mg/dL
16. pH less than 7.1
17. Lactate more than 5 mEq/L
18. Acute thrombocytopenia (<50,000 platelets)
C. Management-based criteria
19. Hysterectomy after infection or hemorrhage
20. Use of continuous vasoactive drugs
21. Cardio-pulmonary resuscitation
22. Dialysis for acute renal failure
23. Any non-anesthetic intubation or ventilation
24. Transfusion of more than 5 units of blood or red cells

^a^Terminology taken from reference [22]

### Data analysis

All of the data were put into a data bank using Microsoft Excel 2007 by two independent researchers. From the maternal outcome, maternal near miss ratio, severe maternal outcome ratio, maternal mortality ratio, maternal near miss: mortality ratio, and mortality index were calculated [12]. To verify the association between the categorical variables and the near miss/death outcome, the chi-square association test or Fisher’s exact test were used (when at least one of these frequencies was less than 5). Crude and adjusted odds ratios were calculated (with 95 % confidence intervals) using logistic regression. To identify independent predictive factors for maternal near miss/ death, a logistical regression model was constructed using a forward stepwise hierarchical selection, including only those variables for which *p* < 0.25 in the bivariate analysis. The Hosmer and Lemeshow test was used to verify the adequacy of the model. The statistical analysis was conducted using Stata® (version 11) software.

### Ethical criteria

The informed consent was waived by Institutional Review Board, since the data were collected directly from medical charts and without interview with patients. The study protocol was approved by the Research and Ethics Committee at the Piauí State University (CAAE 02699812.0.0000.5209).

## Results

During the 6 month period of data collection, 5841 live births, 343 women with severe maternal morbidity, 56 cases of near miss (0.95 % of all deliveries), and 10 maternal deaths (0.2 % of all deliveries) were identified. Cesarean section was the mode of delivery in 58.1 % of control cases, 71.4 % of severe maternal morbidity cases, 87.5 % of near miss cases, and 70 % of maternal death cases. Cesarean sections were stated as elective in 31 cases (22.6 % of severe maternal morbidity cases, 14.3 % of near miss cases and 0 % of maternal death cases) and stated as emergency in 270 cases (77.4 % of severe maternal morbidity, 85.7 % of near miss cases and 100 % of maternal death cases). The main indications of cesarean sections among women with maternal near miss and death were listed as eclampsia/HELLP in 22 %, failed induction in 18.9 %, abruptio placentae in 16 %, obstructed labor in 14.7 %, and fetal distress in 11.5 %. The average length of hospitalization was 8.3 days (4.4 days to severe maternal morbidity cases, 12.5 to near miss and 10.2 to maternal deaths). Table 2 shows that the rate of severe maternal outcomes (near miss + maternal death) was 11.2 cases per 1000 live births. The maternal near miss ratio was 9.6 cases/1000 live births and the mortality ratio was 171.2 cases/100,000 live births (95 % confidence interval 143.3–193.6).

**Table 2:** Obstetric care quality indicators. Teresina, Piaui, Brazil

Indicators	n	Rates
Number of live births	5841	–
Women with severe maternal morbidity	343	–
Women with maternal near miss	56	–
Maternal deaths	10	–
Maternal near miss ratio^a^	–	9.6/1000 LB
Severe maternal outcome ratio^b^	–	11.3/1000 LB
Maternal mortality ratio^c^	–	171.2/100,000 LB
Maternal near miss ratio: maternal mortality ratio^d^	–	5.6:1
Mortality index^e^	–	15.2 %

^a^Maternal near miss ratio: number of cases of near miss/number of live births × 1000

^b^Severe maternal outcome ratio: number of life threatening conditions (near miss + death) / number of live births × 1000

^c^Maternal mortality ratio: number of deaths/number of live births × 100,000

^d^Maternal near miss ratio: maternal mortality ratio

^e^Mortality index: refers “to the number of maternal deaths divided by the number of women with life-threatening conditions expressed as a percentage (number of deaths/number of deaths + number of near miss) [12, 22]

The determinants of severe maternal morbidity, near miss, and maternal deaths are exhibited in Table 3. The main causes were hypertensive (86.1 %), hemorrhagic (10.0 %) and infectious (2.9 %) disorders. Severe pre-eclampsia, eclampsia, and HELLP syndrome were the most frequent determinants of maternal near miss cases. In cases of death the main factors involved were hypertensive complications (40 %), hemorrhagic disorders (30 %) and infectious disorders (30 %). Of all the cases of maternal deaths, 70 % used blood products, 70 % had hysterectomy and 100 % were admitted to Intensive Care Unit. In isolation, infectious abortion was the most common cause of maternal death, responsible for three of the ten deaths (mortality index was highest for abortion, 75 %). Other non-obstetric clinical-surgical conditions were implied in complications for five women.

**Table 3:** Primary determinants of severe maternal morbidity (SMM)^a^, near miss^b^ (NM) and maternal deaths^c^ (MD). Teresina, Piaui, Brazil

Determinants	SMM		NM		MD	
	n	%	n	%	n	%
All cases	343	100	56	100	10	100
Hypertensive disorders^d^						
Severe pre-eclampsia	268	78.1	17	30.4	–	–
Eclampsia	30	8.7	10	17.8	2	20.0
HELLP syndrome	13	3.8	10	17.8	2	20.0
Hemorrhagic disorders^d^						
Abruptio placentae	18	5.2	6	10.7	1	10.0
Uterine atony	2	0.6	3	5.3	2	20.0
Ectopic pregnancy	3	0.9	1	1.8	–	–
Uterine rupture	–	–	2	3.6	–	–
Placenta previa	–	–	2	3.6	–	–
Infectious disorders^d^						
Chorioamnionitis	4	1.2	–	–	–	–
Endometritis	3	0.9	–	–	–	–
Infected abortion	1	0.3	1	1.8	3	30.0
Other clinical conditions-surgeries						
Sepsis after appendicitis	–	–	1	1.8	–	–
Diabetic ketoacidosis	–	–	1	1.8	–	–
Acute pyelonephritis	1	0.3	1	1.8	–	–
Cardiomyopathy	–	–	1	1.8	–	–

^a^Severe maternal complications are defined as “potentially life-threatening conditions” (disease-specific, intervention specific and organ dysfunction-based criteria) [12, 22]

^b^Near miss refers “to a woman who nearly died but survived a complication that occurred during pregnancy, childbirth or within 42 days of termination of pregnancy” [12, 22]

^c^Maternal death is “the death of a woman while pregnant or within 42 days of termination of pregnancy” [12, 22]

^d^Mortality index for the different disease categories: hypertensive disorders (9.7 %), hemorrhagic disorders (17.6 %) and infectious disorders (75 %)

Table 4 shows the cases of maternal near miss and death according to the criteria established by the WHO. All of the cases of maternal death included at least one of the criteria for near miss. Shock was the most common clinical criteria, while the presence of thrombocytopenia was the most frequent of the laboratory criteria. The use of vasoactive drugs, hysterectomy, and transfusion of more than five units of concentrated red blood cells were the most prevalent management criteria.

**Table 4:** Near miss (NM) and maternal deaths according to the WHO criteria. Teresina, Piaui, Brazil

Criteria	NM		Deaths	
	N	%	n	%
All cases	56	100	10	100
Presence of 1 criteria	33	58.9	1	10,0
Presence of 2 criteria	12	21.4	1	10,0
Presence of 3 or more criteria	11	19.7	8	80.0
Clinical criteria^a^				
Shock	12	21.4	4	40.0
Oliguria unresponsive to fluid/diuretics	1	1.8	1	10.0
Loss of consciousness ≥ 12 h	2	3.6	2	20.0
Stroke	1	1.8	–	–
Reentrant seizures	1	1.8	–	–
Laboratory-based criteria^b^				
Oxygen saturation < 90 % for ≥ 60 min	1	1.8	2	20.0
Creatinine ≥ 3.5 mg/dl	3	5.3	2	20.0
Total bilirrubine ≥ 6.0 mg/dl	–	–	2	20.0
Acute thrombocytopenia (<50.000)	10	17.8	2	20.0
Loss of consciousness and presence of glycosuria/ketonuria	1	1.8	–	
Management-based criteria				
Continuous use of vasoactive drugs	11	19.6	6	60.0
Hysterectomy following infection or bleeding	15	26.8	4	40.0
Transfusion of concentrated red blood cells (≥5 units)	10	17.8	3	30.0
Intubation and mechanical ventilation for ≥ 60 min	6	10.7	6	60.0
Dialysis for acute renal failure	2	3.6	1	10.0
Cardio-pulmonary resuscitation	3	5.3	1	10.0

^a^Acute cyanosis, gasping, respiratory frequency > 40 or < 6/min, positive bedside coagulation test, loss of consciousness and absence of pulse, jaundice in the presence of pre-eclampsia were not found

^b^PaO2/FiO2 < 200 mmHg, pH <7.1, lactate > 5 mg/dl were not found

Women in the near miss/death category were more frequently associated with hospitalization of more than 5 days (*p* = 0.023), termination of the pregnancy by cesarean (*p* = 0.002) and APGAR < 7 during the 1^st^ minute (*p* = 0.015). Among the near miss/death cases, there were 20 postpartum hysterectomies (32.3 %) (Table 5). In the multivariate model, only cesarean section (*p* = 0.019) remained as a predictive factor of near miss/death (Table 6).

**Table 5:** Sociodemographic and clinical variables for severe maternal morbidity (SMM) and near miss (NM)/death. Teresina, Piaui, Brazil

Variables	SMM (*n* = 343)		NM/Death (*n* = 66)		CC^a^ (*n* = 824)		SMM vs. CC^b^	NM/Death vs. CC^c^
	N	%	N	%	N	%	OR (CI 95 %)	OR (CI 95 %)
Age < 20 years								
No	266	77.5	49	74.2	654	79.4	1.0	1.0
Yes	77	22.5	17	25.8	170	20.6	1.1 (0.3–1.6)	1.3 (0.5–1.9)
Education < 8 years								
No	209	60.9	41	62.1	505	61.3	1.0	1.0
Yes	134	39.1	25	37.9	319	38.7	1.1 (0.7–1.9)	0.9 (0.6–1.4)
Partner								
Yes	272	79.3	51	77.3	629	76.3	1.0	1.0
No	71	20.7	15	22.7	195	23.7	0.8 (0.5–2.3)	0.9 (0.6–1.8)
Number of pregnancies ≥ 4								
No	295	86.0	57	86.4	722	87.6	1.0	1.0
Yes	48	14.0	9	13.6	102	12.4	1.1 (0.4–1.9)	1.1 (0.6–1.7)
Previous cesarean								
No	89	25.9	21	31.8	264	32.0	1.0	1.0
Yes	254	74.1	45	68.2	560	68.0	1.3 (0.7–2.3)	1.0 (0.8–2.0)
Gestational age < 37 weeks								
No	172	50.6	28	45.2	432	54.5	1.0	1.0
Yes	168	49.4	34	54.8	360	45.5	1.2 (0.6–1.3)	1.5 (0.8–2.3)
Prenatal consultations < 6								
No	165	49.4	31	49.2	402	50.4	1.0	1.0
Yes	169	50.6	32	50.8	396	49.6	1.0 (0.6–1.6)	1.0 (0.7–2.1)
Comorbidities								
No	265	77.3	53	80.3	650	78.9	1.0	1.0
Yes	78	22.7	13	19.7	174	21.1	1.0 (0.5–1.5)	0.9 (0.4–1.9)
Hysterectomy^d^								
No	341	99.4	46	69.7	824	100.0	---	---
Yes	2	0.6	20	30.3	---	---		
Cesarean section (current delivery)								
No	91	27.1	5	8.2	322	40.2	1.0	1.0
Yes	245	72.9	56	91.8	479	59.8	6.2 (1.7–11.3)*	8.3 (1.9–13.1)*
Length of hospital stay (≥5 days)								
No	160	46.6	22	33.3	768	93.2	1.0	1.0
Yes	183	53.4	44	66.7	56	6.8	6.7 (1.5–8.4)*	9.6 (1.7–12.8)*
APGAR 1st minute < 7								
No	277	85.5	39	73.6	744	93.5	1.0	1.0
Yes	47	14.5	14	26.4	52	6.5	2.5 (1.7–4.9)*	4.6 (1.9–5.4)*
APGAR 5th minute < 7								
No	313	96.6	50	94.3	770	96.7	1.0	1.0
Yes	11	3.4	3	5.7	26	3.3	1.0 (0.5–2.6)	1.2 (0.7–2.2)
Fetal weight < 2500 g								
No	195	58.4	29	50.0	445	54.6	1.0	1.0
Yes	139	41.6	29	50.0	370	45.4	0.8 (0.5–1.5)	1.3 (0.8–1.8)
Neonatal death								
No	299	89.5	47	81.0	715	87.7	1.0	1.0
Yes	35	10.5	11	19.0	100	12.3	0.8 (0.4–1.4)	1.4 (0.7–1.9)

*p-value < 0.05 (chi-square association test/Fisher’s exact test)

^a^Control cases

^b^Severe maternal morbidity cases versus control cases

^c^Near miss/deaths cases versus control cases

^d^Criterion for maternal near miss

**Table 6:** Multivariate analysis* of the predictive factors for near miss/death. Teresina, Piaui, Brazil

Variable	OR^a^	OR_adj_^b^	CI 95 %^c^ (OR_adj_)	p-value**
Termination of pregnancy (mode of birth)				
Vaginal	Ref.	Ref.	1.3–16.5	0.019
Cesarean section	4.2	4.2		
APGAR 1st minute < 7				
No	Ref.	Ref.	0.8–3.9	0.183
Yes	2.1	1.7		
Length of hospital stay				
< 5	Ref.	Ref.	0.9–4.0	0.099
5 or more	1.7	1.9		

*Hosmer and Lemeshow test: *p* = 0.471

**Chi-square test

^a^Crude odds ratio

^b^Odds ratio adjusted for all other variables in the table

^c^Confidence interval at 95 %

## Discussion

This is the first hospital-based study about maternal near miss conducted in the city of Teresina, in the Northeast of Brazil. The Dona Evangelina Rosa Maternity is the only unit with obstetric intensive care in the region, serving an at-risk population in an area with around 5 million inhabitants. The study utilized the criteria (clinical, laboratory, and management) proposed by the WHO in 2009, based on the presence of dysfunction or organic failure [12]. The management-based criteria were observed most frequently in the cases of near miss and the only ones indicated in all of the deaths. Other studies also show that the WHO criteria are good markers for identifying maternal near miss due to the fact that they increase the possibility of detection in the most serious cases and in those cases with greater risk for death [16–19]. The near miss cases, since they presented similar characteristics as those of maternal death, can offer important information that can be used to improve obstetric care.

The incidence of maternal near miss in this study (9.6/1000 live births) was higher than that found in hospital-based studies conducted in other states, as Sao Paulo and Sergipe, which reported, respectively, near miss rates of 4.4/1000 live births and 4.7/1000 live births [19, 20]. A systematic review in 2012 showed that the incidence of near miss in Latin America and the Caribbean oscillated between 0.34 and 4.93 %, varying mainly according to the type of criteria used to define the cases [14]. Studies that utilized Waterstone’s criteria, for example, tend to include situations with a lower risk of death and, as such, produce higher rates of near miss [12, 21]. The ratio of maternal mortality for the institution (171.2/100,000 live births) was also higher than that found in Brazil in 2011 (64.8/100,000 live births), which was 164 % higher than the national average [3]. On the other hand, the near miss/death relationship is compatible with data that show that near miss is almost four times more frequent than cases of maternal death; therefore, it is a good parameter for studying cases in which the risk of progressing to death are higher [12–14].

In the present study, the most frequent determinants of severe maternal morbidity were hypertensive disorders, similar to that found in other regions in the country and other developing countries [14, 17, 18, 20]. Nearly 1/4 of maternal deaths in Latin America and the Caribbean are the result of hypertensive complications [10]. In Brazil, a multicentric study in 27 referral hospitals revealed that hypertensive diseases were responsible for 70 % of the cases of severe maternal morbidity, with the ratio of near miss at 4.2/1000 live births [18]. Among the cases of hypertensive complications, 66 % progressed to near miss and 40 % progressed to death, suggesting a delay in the access to care, delayed disease recognition, inadequate or delayed use of antihypertensives and magnesium sulfate. In Brazil, a recent study showed that magnesium sulfate was used in less than 70 % of cases of severe maternal morbidity [18]. Mainly for low-income regions, the reduction of mortality from hypertensive disorders is associated with an improvement in the quality of obstetric care, women’s access to hospitalization, continuing education for healthcare professionals, and a greater availability of beds in intensive care units [10, 22, 23].

Hemorrhagic causes represent 25 % of the near miss cases and 30 % of those that progress to death, with an emphasis on placental abruption and uterine atony. Similar data were found in two other national studies [19, 20]. In Limeira, a city in the Southeast of the country, hemorrhagic complications contributed to 39.5 % of the near miss cases and 40 % of the deaths [19]. While in Aracaju, in the Northeast, the principal determinant of maternal death was hemorrhage in 41.2 % of the cases [20]. A recent multicentric study in 352 health facilities in 28 countries showed that, among the cases of hemorrhagic complications that progressed to a severe maternal outcome (near miss + death), the risk was greater among women who have had less than five years of education, reside in low-income regions, have had more than three pregnancies, had a cesarean, and who had not received prophylactic uterotonic medication [24].

When compared with other causes, abortion was responsible for a small number of cases of severe maternal morbidity and near miss. Nevertheless, it is important to note the fact that in isolation, it was the most frequent determinant (three out of ten) of the cases that evolved to death. Despite the fact that it was not possible to distinguish between spontaneous and induced abortion, infectious complication is a well-known cause of morbidity and mortality when the abortion was induced in an unsafe manner [1, 25]. The frequency of endometritis and sepsis after unsafe abortions has decreased since misoprostol became the predominant method of abortion for Brazilian women [26, 27]. Nevertheless, the use of pills that do not contain the active ingredient or insufficient doses, besides a delay in going to the hospital for fear of being denounced to the police, can contribute to the persistence of hemorrhagic post abortion complications and infections [28, 29]. Young women who do not have a married partner and who have abortions after 14 weeks of pregnancy are at the greatest risk of near miss or death [30].

Other national studies show that women who are older, have less education, have fewer pre-natal consultations, without a married partner, or previous cesarean have the greatest risk of near miss [17–20, 31]. Nevertheless, this was not observed in the present study. The group of women in the maternal near miss/death group only exhibited a greater association with hospitalization greater than 5 days, higher frequency of cesareans in the current pregnancy, and worse neonatal results. The need for interventions to manage severe ill patients can explain a longer period of hospitalization rather than a potential cause of severe morbidity/near miss. Similarly, a greater presence of APGAR < 7 at the 1st minute in the near miss/death group can be understood as a reflection of the greater severity of obstetric illness and of the need to interrupt the pregnancy earlier, a fact which has been observed in other national studies [17–19].

There are controversies in the literature as to whether a cesarean in the current pregnancy can be considered to be a risk for severe maternal morbidity/near miss or, to the contrary, a confounding risk variable. On one hand, interrupting the pregnancy with a cesarean increases the prevalence of infection, hemorrhage, and other complications, which can increase the chance of severe maternal morbidity/near miss [19, 20, 31]. Data from the Ministry of Health show that women who have had a cesarean have 3.5 times greater chance of dying and 5 times greater chance of postpartum infection than those who have a vaginal delivery [32]. On the other hand, the greater frequency of cesareans in this group can be justified by the intrinsic seriousness of near miss cases, which require interrupting the pregnancy earlier to impede the progress of the illness. Another fact that should be recalled is that Brazil has one of the highest rates of cesareans in the world, many times without justification, surpassing half of all deliveries (52 %) in 2010 [32]. In this study, it is possible that high rate of cesarean section was the severe morbidity in itself due to the urgency to resolve the pregnancy, given 89.7 % of them were emergency cesarean sections.

There was a number of limitations to this study that must be considered. First, considering that the Dona Evangelina Rosa Maternity is the only unit in the region that has an obstetric intensive care unit, it is possible that there has been a selection bias. The large concentration of pregnant women with previous comorbidities and obstetric complications might have overestimated the indicators. Secondly, the small number of cases may have contributed why no variables of statistical significance were found to be associated with maternal near miss or death. A future study with more large series could to guarantee statistical power to identify risk factors in the women investigated. Thirdly, the study did not evaluate if the near miss cases occurred due to a delay in seeking medical assistance, difficulty reaching the hospital, or a delay in receiving adequate medical treatment. The near miss development process can be interrupted if the fragility of the system and of health services is recognized [33]. Despite these limitations, one would hope that upon filling in the gaps for the incidence and determinants of severe maternal morbidity and near miss in Teresina, the data from this study can help to implement preventative measures and offer better standards of obstetric care.

## Conclusion

The results show that serious maternal morbidity and near miss affected a large number of women at the Dona Evangelina Rosa Maternity in Teresina during the period in which this study was conducted. In addition, the ratio of mortality encountered was also high, with percentages higher than the national average. Hypertensive diseases were the most frequent cause of serious maternal morbidity and near miss, but infectious abortion was the most common primary isolated determinant of maternal deaths, suggesting that delays may occur in provision appropriate care. Except for cesarean section, no risk factors were identified for near miss. The monitoring of severe maternal morbidity/ near miss cases may allow for evaluation of adequate obstetrical care. The data from the study can be useful to help care providers to recognize early high risk pregnancies and develop interventions for the prevention of severe maternal morbidity.

## Abbreviations

MNM: Maternal near miss
SMM: Severe maternal morbidity
WHO: World Health Organization
OR: Odds ratio
CI: Confidence interval
HELLP: Acronym of the three main features of the syndrome: hemolysis, elevated liver enzymes, and low platelets count
